# Efficacy of preheated chelating agents on calcium ion removal from instrumented root canals

**DOI:** 10.4317/jced.58539

**Published:** 2021-10-01

**Authors:** Meltem Kucuk, Yasar-Meric Tunca, Onur Erdem, Serdar Cetinkaya, Kadriye Demirkaya

**Affiliations:** 1 Near East University, Faculty of Dentistry, Department of Endodontics, Mersin 10 Turkey; 2 University of Kyrenia, Faculty of Dentistry, Department of Endodontics, Mersin 10 Turkey; 3 University of Health Sciences, Faculty of Pharmacy, Department of Pharmaceutical Toxicology, Ankara, Turkey; 4 University of Health Sciences, Faculty of Dentistry, Department of Endodontics, Ankara, Turkey

## Abstract

**Background:**

The heating of chelating agents such as EDTA increases dentin wettability by decreasing surface tension. However, the calcium ion release effect of preheated chelating agents in instrumented root canals has not yet been mentioned. In this study, it was aimed to evaluate the number of calcium ions removed by the pre-heated chelating agents from the root canals.

**Material and Methods:**

After 51 bovine teeth were instrumented, three of them were separated as negative controls and the remaining teeth were divided into six groups according to the temperature of the solution (at 22 or 37ºC): EDTA-22, CITRIC-22, QMix-22, EDTA-37, CITRIC-37 and QMix-37. Following irrigation, calcium ion levels were determined by atomic absorption spectrophotometer in chelating agents collected from the root canals.

**Results:**

QMix solution eliminated significantly more calcium ions than other chelating agents at different temperatures (*p*< 0.05). Regardless of the heating, QMix and 17% EDTA were significantly superior to 40% Citric acid (*p*< 0.05) while no significant difference was detected between QMix and 17% EDTA groups (*p*< 0.05). Heating all chelating agents did not significantly increase their ability to remove calcium ions from pre-instrumented root canals (*P*< 0.05). In the SEM examination, it was observed that the smear layer was removed from the middle third of the roots, except for the negative control group.

**Conclusions:**

Temperature changes have shown that these agents do not increase the ability of the smear layer to dissolve the inorganic structure. QMix at different temperatures may be recommended to use as the final chelating agent.

** Key words:**EDTA, citric acid, QMix, calcium ions, temperature.

## Introduction

During the shaping of the root canal by applying the instruments to the root canal wall, it is inevitable to form smear layers covering the dentin walls and penetrating up to 40 μm depth in the dentinal tubules (1). The smear layer, which may have various thicknesses between <0.5 and 15 μm and blocks the entrance of the dentin tubules, consists of organic and inorganic dental tissue particles, as well as microorganisms and their by-products (2,3). It has been reported that the occurrence of the smear layer can delay or hamper the penetration of antimicrobial agents into the dentinal tubules, and also have a negative impact on the adhesion of sealers to the root canal dentin walls (2,4). For this reason, the smear layer should be completely removed in order to achieve the desired consequences in both the hermetic obturation and disinfection of the root canal system (1,5).

Many agents have been used as an irrigation solution to purify root canals from pulp tissue and debris, and also to eliminate microorganisms (6). Sodium hypochlorite (NaOCl), which has great antimicrobial potential, is mostly used during the instrumentation of the root canal system and can act only on the organic components of the smear layer (7). Therefore, the use of various chelating agents such as citric acid and Ethylenediaminetetraacetic acid (EDTA), are recommended for the final irrigation of the root canal system to dispose of the inorganic components of the smear layer on the root canal walls (8). In addition, QMix 2in1 (Dentsply Tulsa, Maillefer, Ballaigues, Switzerland) has been recently developed by adding detergents and chlorhexidine to EDTA (accepted as a gold standard) and became another recommended chelating agent to be used (1).

One of the advantages of QMix compared to EDTA and citric acid is to have a surface-active agent as an auxiliary substance that facilitates the wetting of the root canal wall and penetration of irrigants through the dentin tubules by decreasing its surface tension (9). However, the surface tension, which is the condition of intermolecular attraction, can be reduced not only by the addition of surfactant but also by the application of heat (10). In line with this information, in this study, it was primarily aimed to enlighten whether QMix has a superiority compared to 17% EDTA and 40% citric acid solutions in terms of surface tension regardless of their temperature. Secondly, it was aimed to compare the effect of temperature changes on the surface tension of these chelating agents by evaluating the number of calcium ions dissolved in the smear layer.

## Material and Methods

Mandibular incisors were extracted from cattle aged three years old and put in a 1% thymol solution after periodontal tissues removal under a protocol approved by the Animal Research Ethics Committee, Near East University (ID: 2020/115). Forty-eight bovine mandibular incisors with mature apices and similar buccolingual and mesiodistal dimensions at the cementoenamel junction were selected and had their coronal parts removed at a distance of 18 mm from the apex for standardization. All standardized roots (10 ± 1 mm of mesiodistal and buccal-lingual root diameter) were examined under the light microscopy (Leica DMIL, Leica Microsystems Wetzlar GmbH, Wetzlar, Germany) to ensure the absence of defects, cracks or fractures before experiment. Pulp tissue extirpation was accomplished with the aid of a #25 barbed broach and then the estimated working length was established introducing a #10K-file into each canal until its tip was just seen at the apical foramen. The real working length was ascertained as 1.0 mm shorter than this length. Each root canal was shaped with ProTaper-Universal instruments (Dentsply-Maillefer, Ballaigues, Switzerland) up to apical size F5, and 2 mL of 5.25% NaOCl (Cerkamed, Stalowa Wola, Poland) was used as an antimicrobial solution between each instrument during cleaning and shaping. A rinse of 5 mL distilled water was performed for removing of residual NaOCl solution and canals were dried using ProTaper F5 paper points (Dentsply, Konstanz, Germany). The external surfaces of all roots were covered with two layers nail polish to inhibit undesired demineralization caused by overflow solution and apical openings were also sealed with cavit.

After canal preparation procedure, the forty-eight roots were randomly divided and named into six groups (n = 8) according to final chelating agent and temperature, as followed.

Group EDTA-22; 17% EDTA (Cerkamed, Stalowa Wola, Poland) at 22ºC.

Group CITRIC-22; 40% citric acid (Cerkamed, Stalowa Wola, Poland) at 22 C.

Group QMix-22; QMix at 22ºC.

Group EDTA-37; 17% EDTA at 37ºC.

Group CITRIC-37; 40% citric acid at 37ºC.

Group QMix- 37; QMix at 37ºC.

Three roots were used as a negative control group for final irrigation only with distilled water. The 40% citric acid, 17% EDTA and QMix solutions which had been stored at room temperature (22ºC) were heated in a heating cup until the gauge of Digital Thermometer (HAACP Digital Thermometer, UK) shows body temperature (37ºC). The lid of 15-mL Falcon tube was perforated and thus the coronal part of the root could be fixed outside, leaving the rest in tube apically. In each canal, a 27-gauge needle was placed as one mm shorter than working length and final irrigation was performed for one minute. The total volume of final chelating agent which was delivered through the entire canal and exiting through the coronal patency into the collection tube was 5 mL per root. After the chelating agents were collected in tubes (one tube per root), each root was separated from the tubes and removed. All tubes were forwarded to Atomic Absorption Spectrophotometer (AAS) for quantification of calcium ion concentration within the solution following the replacement of new labelled lids.

Besides, one root from each group was randomly selected in order to observe the remaining smear layer under scanning electron microscopy (SEM). Immediately after the final irrigations were completed, the root canals were re-dried with paper tips, and then longitudinal grooves were created on the buccal and lingual surfaces of each root using diamond discs. A cement spatula was placed in the grooves and the roots were split longitudinally into mesial and distal segments with the help of a hammer. Afterward, only one-half of each root was selected for imaging. For dehydration, half of each root was stored in increasing concentration of ethanol solutions (70, 80 and 100%), respectively. The canal surfaces of the roots were then coated and photographed by SEM at ×1000 magnification.

-Determination of Calcium (Ca2+) Levels

Ca2+ levels in pre-treated samples were measured using an Atomic Absorption Spectrophotometer (Perkin Elmer Analyst 800, MA, USA). Flame AAS was used in all process and Air/Acetylene was used to produce flame in this method. 1 g/L of Ca2+ standard solution (Merck) was diluted to prepare dilutions for the standard curve and calibration purpose. C-HCL type hollow cathode lamp which is specific for Ca element was used in the analysis. Method parameters such as wavelength, slit width, signal type, signal time, sample volume, lamp stream was given in Table 1. Calibration curve was drawn for 1, 2, 4, 8, 16 and 32 μg/ml Ca2+ standard solutions (R2= 0.999) (Table 1).

**Table 1:**
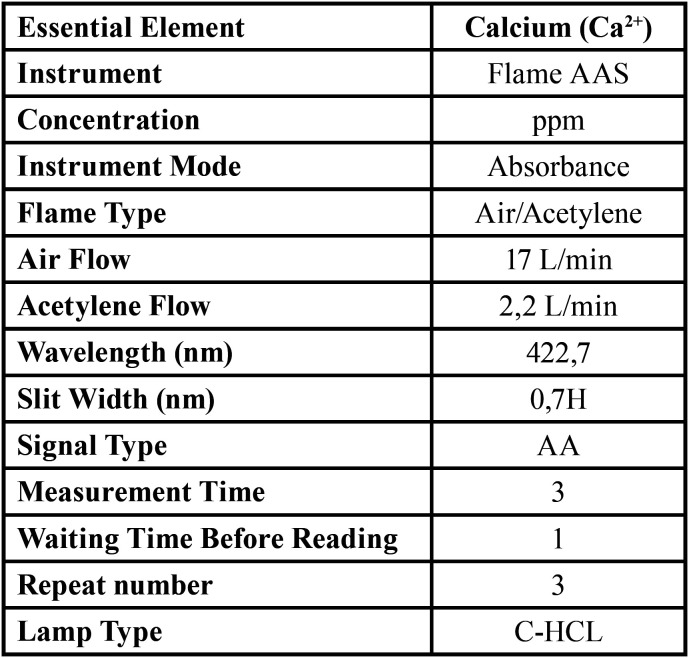
AAS operating parameters.

-Statistical analysis 

The normal distribution of the groups was examined with the Shapiro-Wilk Test, and since it was observed that they match the normal distribution (*p*> 0.05), parametric one-way analysis of variance ANOVA was used. To reveal which group the difference originated from, post-hoc Tukey’s HSD test was performed. Comparisons of two independent groups were tested with the Student’s t test.

## Results

**Figure 1:**
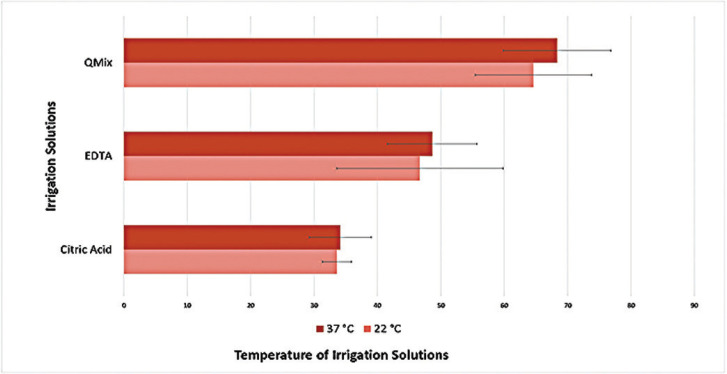
The percentage (%) of calcium ions removed by various chelating agents at different temperatures.

**Figure 2:**
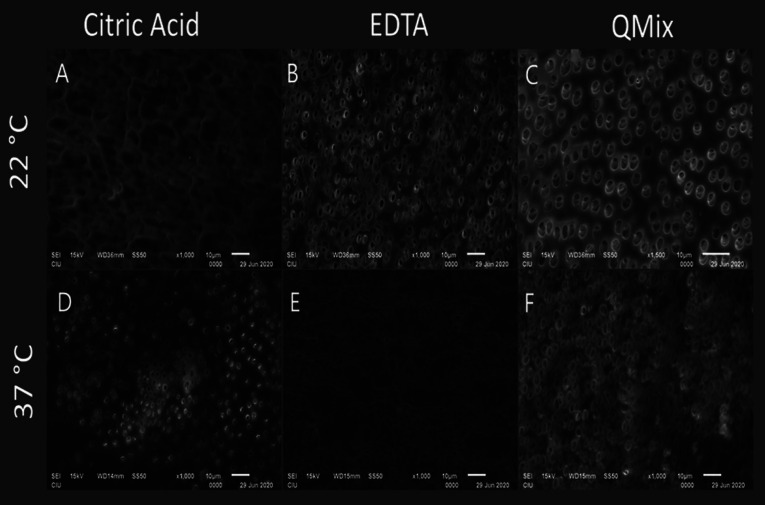
Representative scanning electron microscope images of the middle third of the roots from each group. (A, E) The irregular dentin surface is shown, which makes it somewhat difficult to identify dentinal tubules. However, the smear layer was mostly removed. (C) Regular and smooth dentin surface without smear layer is visible (B, D, F) Chelating agents show similar efficacy in removing smear layer. There is no smear layer covering the dentinal tubules.

**Figure 3:**
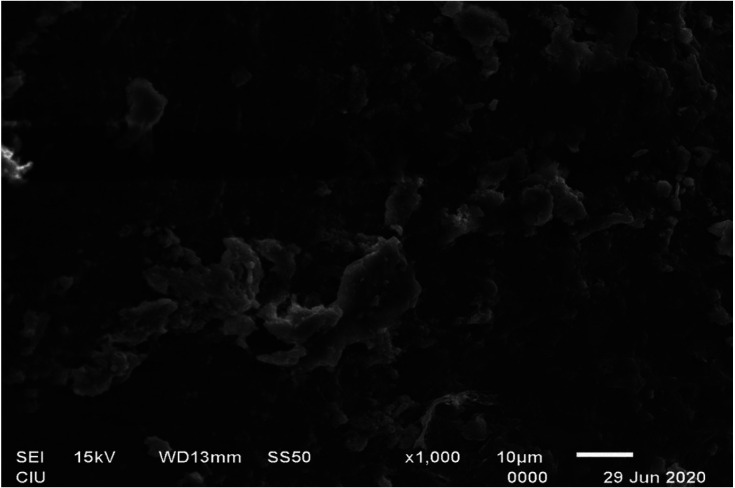
Representative scanning electron microscopy image of a root canal surface in negative control group at the middle thirds. Thick homogeneous smear layer covers the entire canal surface.

It was observed that QMix eliminated significantly more calcium ions than EDTA (ANOVA, Tukey *p*<0.05), and EDTA eliminated significantly more calcium ions than CITRIC in both 22 and 37 temperatures (ANOVA, Tukey *p*<0.05). When the solutions were compared regardless of the temperature QMix and EDTA were significantly superior to CITRIC (ANOVA, Tukey *p*<0.05), while QMix and EDTA were similar in terms of calcium ion removal (ANOVA, Tukey *p*>0.05) (Table 2, Fig. 1). When the solutions were compared for different temperatures, no significant difference was found between different temperatures for all three solutions (t-test, *p*>0.05). In the examination of the images obtained with SEM, apart from the negative control group, it was observed that the smear layer was removed from the middle thirds of roots (Fig. 2,3).

**Table 2:**
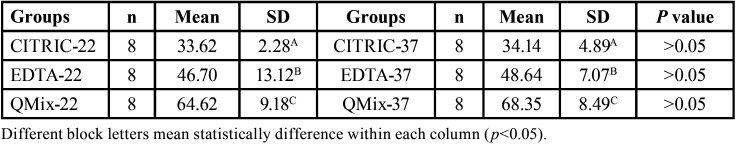
Calcium ion elimination values (ppm) from root canal dentin following 1-minute irrigation in intra-group and inter-groups.

## Discussion

Removal of the smear layer has a vital role in successful root canal treatment for sanitization and hermetic filling of the root canal system (11). Although various methods of irrigation (laser, ultrasonic and sonic, etc.) and chemical agents are used to remove the smear layer, none of them completely removes the smear layer from the root canal system (12). This may be due to the inadequacy of chemical solutions used to wet the root canal dentin surface and penetrate through the dentin. The optimal wettability of root canal walls depends on the surface tension of the chemical solution used and the surface energy of root canal dentin (2). Preheating the chemical solution reduces surface tension and increases tissue dissolving properties without any change in their short-term stability (13,14). Higher temperature may enhance surface wetting ability and penetration of solutions into the dentin tubules, as well as the effect of killing the endodontic microbiota (15). Besides, a lower concentration solution at a high temperature may be less toxic compared to high-concentration at low temperature (13,16). Yılmaz *et al*. found that when the temperature of EDTA solution was increased from 22ºC to 37ºC, its surface tension decreased dramatically, and on account of this finding, at different temperatures were employed in our study (10). The effects of temperature changes of 17%EDTA, 40% citric acid and QMix agents on dissolving the smear layer were evaluated by calcium ion measurement and the effectiveness of these chelating agents was shown not to be notably affected by the temperature increase in this study.

Present analysis of calcium ion concentrations dissolved by chelators from the smear layer showed that QMix removes more smear layer compared to 17%EDTA and 40%citric acid in the body and room temperature. These findings agree with the results of previous studies showing that QMix removes the smear layer better than EDTA (17,18). The addition of auxiliary substances such as surfactant or chlorhexidine to the EDTA significantly increases the wettability of the root canal dentin surfaces and also leads to an increase in the bactericidal activity of the chelating agent (19,20). Additionally, QMix (containing EDTA, CHX and surfactant) may provide better and long-term antimicrobial activity penetrating into dentin tubules due to retaining of chlorhexidine in the dental hard tissue up to 120 days (9,20). Unlike our findings, it was also found in previous studies that EDTA and citric acid had statistically the same ability in terms of removing the smear layer with activation. The difference between the results may be due to the fact that activating a chelator allows further debridement along the root canal and the occurrence of the phenomenon known as flow hydrodynamics (21,8,22). Another reason for this discrepancy in the results may be the use of the rating system developed by Hülsmann *et al*. in the methodology, unlike ours (2,23). In the literature, SEM is one of the most widely used techniques to assess the presence of the smear layer, but the SEM examination limits this evaluation by the inability to distinguish the smear layer from the sclerotic dentine, especially at apical third (24,25). Therefore, in this study, AAS was used to confirm the removal of the smear layer by determining the number of calcium ions in 17% EDTA, 40% citric acid and QMix solutions. SEM observation was utilized only as a supplement to confirm that the smear layer was removed. Also, bovine teeth at the same age and similar sizes were used as a specimen to standardize the dentin surface properties as much as possible.

Chelation agents can remove minerals from the dentin tissue while demineralizing the smear layer and cause an undesired effect on the chemical and physical properties of dentin tissue such as microhardness (26). However, there is no consensus in the literature about the recommended application times in the canal to avoid the side effects of chelation agents on root dentine (22). Irrigation protocol was done in the canal for one minute, considering that one-minute application of 17% EDTA and 10% citric acid did not make a notable difference in the microhardness of dentin according to De-Deus *et al*. (27). In addition to this, after removing the smear layer by using a chelating agent, it is reasonable to irrigate the canal again with another disinfecting solution to attack the bacteria remaining in the dentin tubules (28). However, when NaOCl is used as a final disinfectant for this purpose, it shows the corrosive effect on the dentin microstructure. Therefore, using QMix as a single final solution and thus reducing disinfection steps can provide optimal final irrigation without dentin erosion caused by NaOCl (1,28).

Unexpectedly, the results of the present study showed that 17%EDTA, 40% citric acid and QMix solutions have not eliminated more Calcium ions at 37ºC compared to 22ºC. Similar to these results, Keskin and Çiçek reported in their study under scanning electron microscopy that EDTA at 37ºC did not significantly remove the smear layer compared to 25ºC. Therefore, more clinical studies are needed to reveal whether the temperature increase alters the surface tension property of the chelating agents and removes more of the smear layer.

## Conclusions

The current study has examined the degree of elimination of calcium ions from smear layers by various chelating agents at different temperature and temperature changes have indicated that these agents increase the ability in order to dissolve the inorganic structure of the smear layer. All things considered, QMix can be recommended to use as the final chelating agent in terms of dissolving the smear layer better than 17% EDTA and 40% citric acid at both temperatures (22 and 37 0C). Nonetheless, differently designed studies are needed to clarify the effect of pre-heated chelating agents on the root dentin microstructure, the adhesion of root canal sealers or antimicrobial activity inside the root canal system.
